# Nanomedicines: Emerging Platforms in Smart Chemotherapy Treatment—A Recent Review

**DOI:** 10.3390/ph17030315

**Published:** 2024-02-28

**Authors:** Mosab Arafat, Molham Sakkal, Rami Beiram, Salahdein AbuRuz

**Affiliations:** 1 College of Pharmacy, Al Ain University, Al Ain P.O. Box 64141, United Arab Emirates; mosab.arafat@aau.ac.ae (M.A.);; 2 Department of Pharmacology and Therapeutics, College of Medicine and Health Sciences, United Arab Emirates University, Al Ain P.O. Box 17666, United Arab Emirates; 3 Department of Biopharmaceutics and Clinical Pharmacy, School of Pharmacy, The University of Jordan, Amman 11942, Jordan

**Keywords:** cancer, chemotherapeutic drugs, controlled drug release, drug delivery systems, nanomedicine, passive and active drug delivery, selective targeting

## Abstract

Cancer continues to pose one of the most critical challenges in global healthcare. Despite the wide array of existing cancer drugs, the primary obstacle remains in selectively targeting and eliminating cancer cells while minimizing damage to healthy ones, thereby reducing treatment side effects. The revolutionary approach of utilizing nanomaterials for delivering cancer therapeutic agents has significantly enhanced the efficacy and safety of chemotherapeutic drugs. This crucial shift is attributed to the unique properties of nanomaterials, enabling nanocarriers to transport therapeutic agents to tumor sites in both passive and active modes, while minimizing drug elimination from delivery systems. Furthermore, these nanocarriers can be designed to respond to internal or external stimuli, thus facilitating controlled drug release. However, the production of nanomedications for cancer therapy encounters various challenges that can impede progress in this field. This review aims to provide a comprehensive overview of the current state of nanomedication in cancer treatment. It explores a variety of nanomaterials, focusing on their unique properties that are crucial for overcoming the limitations of conventional chemotherapy. Additionally, the review delves into the properties and functionalities of nanocarriers, highlighting their significant impact on the evolution of nanomedicine. It also critically assesses recent advancements in drug delivery systems, covering a range of innovative delivery methodologies. Finally, the review succinctly addresses the challenges encountered in developing nanomedications, offering insightful perspectives to guide future research in this field.

## 1. Introduction

Cancer is the second leading cause of death worldwide, accounting for approximately one-sixth of all deaths. In 2022, there were 20 million new cancer cases and approximately 10 million cancer deaths globally [1]. Current approaches to cancer diagnosis and treatment utilize established screening techniques tailored for specific cancer types, followed by interventions such as surgery, radiation, chemotherapy, targeted therapy, and immunotherapy [2].

Although chemotherapy remains the most efficient and cost-effective choice for many patients, especially those with advanced diseases, ongoing research has developed other promising cancer treatments, such as immunotherapy, hormone therapy, gene therapy, and stem cell therapy [3]. Despite notable advancements in both biological and clinical aspects, resulting in improved outcomes for cancer patients, the majority of modern chemotherapeutic agents still cause undesirable and severe side effects [4].

Chemotherapy drugs work by interfering with different stages of cell division, allowing them to kill rapidly dividing cancer cells. However, the non-specific nature of these drugs necessitates high doses for effectiveness [5]. This lack of selectivity can affect rapidly dividing healthy cells, resulting in side effects such as nerve damage, nausea, general discomfort, bone marrow suppression, hair loss, kidney damage, and heart damage [3]. Additionally, several chemotherapy drugs are insoluble in water, posing challenges in formulation and absorption. Another challenge is the development of cancer cell resistance to these drugs over time [6].

Nanotechnology has revolutionized drug delivery in various medical fields. In ophthalmology, nanomedications like Restasis, Cequa^®^ [7], and Cyclokat^®^ [8] address dry eye syndrome. Durezol combats eye inflammation [9], while Ikervis^®^ is used for acute keratitis [7]. For neurodegenerative conditions, treatments include Riluzole nanoparticles for amyotrophic lateral sclerosis [10], and Glatopa^®^ for multiple sclerosis [11]. Nanomedications such as DepoDur™ [12], and Exparel^®^ [13] have enhanced pain management. The COVID-19 vaccines from Pfizer–BioNTech and Moderna, utilizing lipid nanoparticles for mRNA delivery, underscore nanotechnology’s pivotal role in enhancing vaccine delivery [14]. Cancer therapy remains a major area of focus, with numerous nanomedications approved, demonstrating the breadth of nanotechnology’s application in modern medicine [15].

The use of nanoparticles has been shown to improve the solubility of poorly soluble drugs, making them more easily absorbed by the body [16,17]. Nanocarriers have become essential tools in cancer therapy, utilizing the enhanced permeability and retention (EPR) effect. Due to the high vascular permeability and reduced lymphatic outflow in solid tumors, the EPR effect enables precise passive targeting and prolonged retention of nanoparticles at the tumor site. As a result, nanomedicines significantly enhance therapeutic outcomes while minimizing the dose-dependent toxicity associated with traditional chemotherapy [18,19].

In recent advancements, next-generation nanocarriers have demonstrated smart strategies for targeted cancer cell delivery and improved treatment results. These innovative approaches encompass ligand-based active tumor targeting and tumor microenvironment (TME) responsive drug delivery [20]. Ligand-based targeting involves the direct interaction of nanocarrier ligands with overexpressed receptors or antigens on cancer cells, leading to the selective uptake of nanocarriers. Additionally, TME-responsive delivery systems release drugs selectively in response to the unique physiological features of the TME, distinguishing them from healthy tissues [21,22].

**Figure 1:**
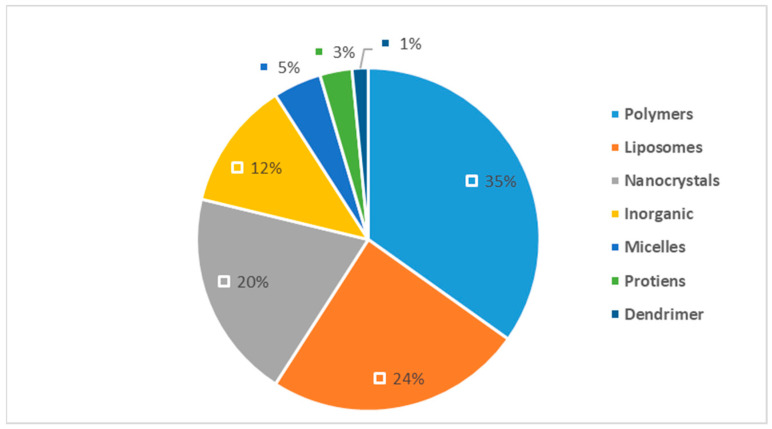
Distribution of nanoparticle types in nanomedicine [26,27,28,29,30,31,32,33].

The distinctive targeting ability of nanoparticles is attributed to unique characteristics found in various types of nanomaterials. These materials consist of tiny particles with large surface areas, enabling the controlled release of drugs and their precise delivery to specific targets. In the field of cancer therapy, a diverse array of nanomaterials has been employed to develop nanomedications, including lipid-based nanoparticles, polymers, nanocrystals, nanoproteins, and inorganic nanoparticles [23,24,25]. Figure 1 illustrates the different types of nanoparticles and their respective proportions used in the development of approved nanomedications for clinical use. Appendix A provides the full list of these approved medications [26,27,28,29,30,31,32,33].

Approved nanomedications for cancer therapy possess several advantages due to the incorporation of nanotechnology. For instance, liposomal nanoparticles used in the formulation of Doxil significantly reduced the systemic toxicity of free drugs and enhanced drug delivery to the site of the tumor [15]. Similarly, Myocet, employed in the treatment of metastatic breast cancer, utilized liposomal nanoparticles to achieve improved drug delivery [34]. Another study on the Eligard anticancer medication used a nanocarrier comprising leuprolide acetate and the polymer PLGA (poly(DL-lactide-co-glycolide)), ensuring controlled and extended delivery of the therapeutic payload [30]. Additionally, advancements in drug solubility were achieved with drugs such as paclitaxel, utilizing albumin-bound nanoparticles. This formulation has been proven effective in treating breast cancer, non-small-cell lung cancer, and pancreatic cancer [35].

**Figure 2:**
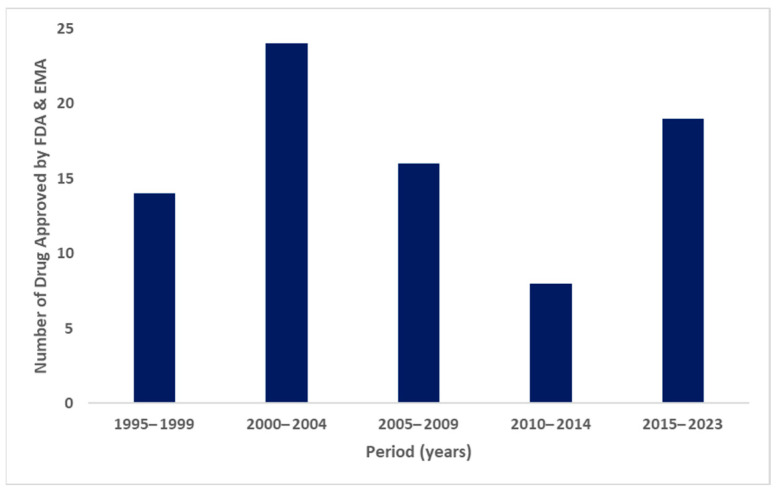
FDA-approved nanomedicines in previous years [26,27,28,29,30,31,32].

Despite nearly three decades having passed since the approval of the first nanomedication for cancer treatment, and despite significant efforts in developing new nanomedications for cancer therapy, the annual approval rate of these medications has remained relatively constant. This trend is illustrated in Figure 2, which presents the FDA-approved nanomedications [26,27,28,29,30,31,32]. The slow progress can be attributed to several barriers encountered in the development of nanomedications, including the need to devise effective and scalable manufacturing processes [26,36]. Additionally, it is necessary for regulatory agencies to establish new guidance and standards for their evaluation [37]. Furthermore, not all nanomaterials have undergone extensive testing in humans, posing a potential risk of toxicity associated with their usage [38].

This research review aims to provide a comprehensive overview of the current state of nanomedication in the treatment of cancer. This review demonstrates the various types of nanomaterials and their properties in overcoming the limitations of conventional chemotherapy. It also discusses the diverse properties and characteristics of nanocarriers and their influence on the development of nanomedicine, along with the latest advances in smart drug delivery systems and various delivery methods. Finally, it briefly covers the obstacles encountered in developing nanomedications.

## 2. Types of Nanomaterial

Different types of materials have emerged in the field of nanoparticles, each with its advantages and limitations. Researchers and scientists have invested significant effort in enhancing existing nanomaterials, resulting in several new generations of nanomaterials emerging in the last decade to overcome the limitations of conventional ones. The following sections provide a comprehensive overview of the most used nanomaterials, along with their latest advancements in the field of cancer.

### 2.1. Lipid-Based Nanoparticle

Lipid-based formulations have been extensively studied for their potential in cancer therapy [20,39]. These formulations are biocompatible, biodegradable, and enhance drug efficacy. They can encapsulate both hydrophilic and hydrophobic drugs, in addition to having a high drug-loading capacity. They also release drugs slowly and in a controlled manner. Additionally, they can be tailored to possess specific properties that influence their behavior in the body [39,40]. Lipid-based formulations can be categorized according to their composition and physicochemical properties into three main groups: liposomes [41], solid lipid nanoparticles (SLNs) [42], and nanostructured lipid carriers (NLCs) [43]. Table 1 demonstrates different studies on lipid-based nanoparticles.

#### 2.1.1. Liposomes

Liposomes exhibit a diverse range of classifications based on their surface charge (neutral, negative, or positive) and structural characteristics, including the number of lipid bilayers and vesicle size. Structurally, liposomes can be categorized into multiple types. Unilamellar liposomes (ULVs) consist of a single lipid bilayer, and they can be further distinguished by their size: small unilamellar vesicles (SUVs) measuring between 20 and 100 nm, large unilamellar vesicles (LUVs) exceeding 100 nm, and giant unilamellar vesicles (GUVs) with sizes above 1000 nm [55]. On the other hand, multilamellar liposomes (MLVs) feature multiple lipid bilayers and typically have a size greater than 500 nm. Additionally, multivesicular liposomes (MVVs) represent a specialized subcategory of multilamellar liposomes, containing smaller unilamellar vesicles within their bilayers, and they typically exceed 1000 nm in size. These vesicles can encapsulate hydrophilic agents within the aqueous core and hydrophobic agents within the lipid bilayer [42,55].

Liposomes are recognized as safe and versatile nanocarriers, primarily because they are composed of biocompatible and biodegradable lipids like phospholipids and cholesterol [56]. Additionally, liposomes can be easily tailored with different compounds to achieve various objectives, such as prolonging their circulation in the bloodstream, enhancing their ability to target specific sites, facilitating cellular uptake, and enabling controlled drug release in response to specific stimuli [57,58]. These favorable characteristics have formed the foundation for the successful application of liposomes in clinical settings. Currently, several liposomal formulations are in regular clinical use, and many others are progressing through different stages of clinical trials or awaiting regulatory approval [39,59].

Conventional liposomes are used to reduce the side effects of chemotherapy drugs by altering their distribution in the body and delivering them more specifically to tumor tissues. However, these liposomes are quickly cleared from the bloodstream, which limits their effectiveness. The primary reason for the rapid clearance of liposomes from the bloodstream is a process known as opsonization. In this process, opsonins bind to the liposomes, making them more recognizable and easily engulfed by the reticuloendothelial system (RES) [60]. Stealth liposomes have been developed by incorporating hydrophilic polymers, such as polyethylene glycol (PEG), as steric stabilizers. PEG coatings effectively shield the liposome surface, preventing aggregation, opsonization, and phagocytosis, thus extending their systemic circulation. This enables stealth liposomes to remain in the bloodstream for a longer period, facilitating the more efficient delivery of anticancer drug molecules to tumor tissues [61,62].

Nanoparticles made of liposomes are widely used in cancer therapy due to their high selectivity for tumor cells. In healthy tissues, liposomes typically remain within the bloodstream because of the presence of tight junctions between endothelial cells that prevent the leakage of particles from the blood vessels. Conversely, tumor vessels exhibit higher permeability compared to those in healthy tissues, enabling nanoscale liposomes to exit the bloodstream, penetrate the tumor tissue, and release the drug molecule [59,63].

Lately, new strategies have emerged to enhance tumor cell selectivity, reduce side effects, and boost drug efficacy. These include selectively binding to receptors or molecules overexpressed in cancer cells and within the tumor microenvironment. This precise targeting ensures accurate drug delivery directly to tumors. A variety of ligands, such as antibodies, proteins, peptides, vitamins, growth factors, and aptamers, can be used to target liposomes to specific cells [20,44]. For instance, the attachment of anti-CD22 monoclonal antibodies to PEGylated liposomes loaded with doxorubicin (DOX) enhances drug accumulation in non-Hodgkin’s lymphoma tumors [44]. In another study, paclitaxel liposomes designed to target the folate receptor (FR) demonstrated improved efficacy in eradicating cancer cells and a prolonged half-life in the body when compared to non-targeted liposomes. The transferrin receptor (TfR) represents another valuable molecule for liposome-targeting cancer cells [20]. In another study performed in a mouse model involving colon cancer, TfR-targeted liposomes (Tf-PEG-liposomes) exhibited extended circulation in the bloodstream and reduced susceptibility to uptake by the RES. This resulted in increased liposome accumulation in tumor tissue, thus enhancing drug delivery to cancer cells via endocytosis [45].

Moreover, liposomes can be engineered for drug release in response to the tumor microenvironment. For example, folate-linked pH-responsive liposomes delivering irinotecan displayed pH-dependent sustained drug release, enhanced tumor cell uptake, and superior efficacy against colorectal cancer in comparison to pH-sensitive systems or free irinotecan [15].

Liposome nanoparticles hold promise for various unmet medical needs beyond oncology. For instance, in 2023, researchers developed liposomes functionalized with the neurofilament-derived peptide, NFL-TBS.40–63, specifically to penetrate the blood–brain barrier (BBB) and target glioblastoma cells. This NFL peptide not only facilitated the crossing of the BBB but also enhanced the liposomes’ internalization into glioblastoma cells [46]. Additionally, several liposomal nanoformulations for cancer therapy have progressed to clinical stages. Notably, siRNA targeting EPHA2 with liposomes for advanced neoplasm is currently in Phase I trial recruitment under the proprietary name siRNA-EPHA2-DOPC (2012) [47], while liposomal paclitaxel for breast cancer has reached Phase III trial stages (EndoTAG-1, 2016) [48]. HER2-targeted liposomal DOX hydrochloride for breast cancer underwent a Phase III trial that was terminated (MM-302, 2014) [49], and thermally sensitive liposomal DOX for breast cancer completed Phase III trials (ThermoDox, 2009) [15].

#### 2.1.2. Solid Lipid Nanoparticles (SLNs)

SLNs consist of a solid lipid core with a surfactant layer designed to enhance their stability in aqueous environments. The lipid materials used in SLN formulation encompass a diverse range, including triglycerides (such as tristearin, tripalmitin, and trilaurin) [64], partial glycerides (like glyceryl behenate, glyceryl stearate, and glyceryl palmitostearate), fatty acids (including stearic acid, palmitic acid, and capric acid) [42], steroids (such as cholesterol), and waxes. Commonly used surfactants comprise poloxamers, lecithin, sodium glycocholate, polysorbates, sorbitan esters, and their mixtures [65]. SLNs offer protection to encapsulated drugs against chemical degradation and enable controlled drug release. These nanoparticles create stable nanosuspensions with longer-lasting effects compared to liposomal delivery systems. Furthermore, SLNs are versatile and can be administered via various routes, including dermal, oral, pulmonary, and parenteral [66].

Several studies have demonstrated the potential of SLNs to enhance the efficacy and safety of various anticancer drugs. Ongoing research in this field is promising [50,51]. SLNs loaded with gemcitabine were tested on patient-derived primary pancreatic cancer cell lines (PPCL-46) and MiaPaCa-2 pancreatic cancer cell lines. The study revealed that SLNs can deliver gemcitabine to pancreatic cancer cells more effectively than gemcitabine alone, suggesting that SLNs could be a promising new approach to treating pancreatic cancer [50]. In another study, researchers prepared SLNs conjugated with transferrin to deliver tamoxifen citrate for breast cancer treatment. The enhanced targeting capability of tamoxifen citrate against MCF-7 breast cancer cells provided evidence of the potential effectiveness of SLNs for breast cancer therapy [51].

A new potential for breast cancer treatment has emerged with novel SLNs modified using arginine-glycine-aspartic (RGD) tripeptides to enhance breast cancer cell targeting. These pH-sensitive SLNs release DOX in the acidic TME. In vitro, they demonstrated superior efficacy in eliminating breast cancer cells compared to DOX alone. In vivo, they effectively reduced tumor size and improved survival rates in mice with breast cancer. These promising results suggest that these innovative SLNs could offer a new approach to breast cancer treatment. They are currently in preclinical development, with plans for future clinical trials [52].

#### 2.1.3. Nanostructured Lipid Carriers (NLCs)

NLCs represent the second generation of lipid nanoparticles, specifically designed to address the limitations observed in SLNs. These limitations encompass issues such as a restricted drug-loading capacity, polymorphic transitions, the tendency of lipids to crystallize over time, and the potential for drug leakage during storage [67]. In general, NLC formulations consist of a combination of solid and liquid lipids, surfactants, and various additional components, including co-surfactants and counterions [68]. In this composition, a solid lipid matrix is embedded in a liquid lipid phase. The addition of liquid lipids transforms the solid lipid matrix from a highly structured crystalline state to a less ordered crystalline lattice. This transformation plays a crucial role in increasing drug-loading capacity while simultaneously preventing unwanted drug leakage [68,69].

A recent study encapsulated the anticancer agent harmaline in folate-chitosan-coated nanostructured lipid carriers (FCH-NLCs) to evaluate their effect on MCF-7 breast cancer cells. These FCH-NLCs, measuring 153 nm, significantly reduced cell survival by triggering the overexpression of apoptotic genes (Caspase 3, 8, 9) in the MCF-7 cells, leading to apoptotic cell death. Additionally, the FCH-NLCs selectively targeted cancer cells, likely through folate receptors, making them a promising option for treatment [53]. Another research team successfully optimized nanostructured lipid carriers (NLCs) loaded with metformin and thymoquinone. These NLCs, measuring just 78.99 nm and achieving high drug entrapment efficiencies of 90% for metformin and 91% for thymoquinone, exhibited superior efficacy in eliminating breast cancer cells. They not only induced apoptosis but also caused cell cycle arrest, outperforming the individual drugs in effectiveness [54].

### 2.2. Polymers

Polymers can be derived from natural sources, such as proteins, peptides, and cellulose, or obtained synthetically, such as poly lactic-co-glycolic acid, poly(glycolic acid), and polyethylene glycol, or they could be pseudosynthetic [70,71]. Polymers are widely used in the field of nanomedication for several reasons. These include ease of synthesis, high stability, the ability to control drug release, biodegradability, low immunogenicity, low toxicity, and high entrapment efficiency, making them suitable for scaling up. Polymers can assume various forms, such as polymer–drug conjugates, polymeric micelles, and dendrimers [70,72,73,74].

#### 2.2.1. Polymer–Drug Conjugates

In polymer–drug conjugates, the therapeutic agent is covalently linked to a hydrophilic polymer through biologically active linkers. Various types of polymers can be utilized, including PEG, poly(L-glutamic acid), polystyrene-maleic anhydride copolymer, and N-(2-hydroxypropyl) methacrylamide [70]. This system offers several advantages, including drug delivery to the target site, reduced side effects, increased drug loading, and controlled drug release [70,75].

Various medications have been developed into polymer–drug conjugates to enhance their pharmacokinetic properties. An example is Pegaspargase (Oncaspar), which conjugates the chemotherapy agent asparaginase with the polymer PEG. Approved in 1994 for treating acute lymphoblastic leukemia, Pegaspargase has a significantly longer half-life of 357 h compared to the 20 h of L-asparaginase. This extended half-life allows for less frequent administration to patients [76]. Another example is antibody fragments, which are smaller and easier to produce than full antibodies but have a shorter half-life. PEGylation can address this limitation, as demonstrated by certolizumab pegol, a PEGylated antibody fragment with a half-life of 14 days, allowing for biweekly administration [77].

#### 2.2.2. Polymeric Micelles

Polymeric micelles consist of a hydrophobic core and a hydrophilic shell, forming through the self-assembly of amphiphilic block copolymers in water. The hydrophobic core enables the storage of poorly water-soluble drugs, thereby increasing their bioavailability. Meanwhile, the hydrophilic shell enhances micelle stability in the bloodstream, extending their circulation time [78,79]. To enhance tumor targeting, tumor-specific ligands can be attached to polymeric micelles, making them a promising platform for delivering hydrophobic drugs for cancer treatment [80]. One notable advantage of polymeric micelles over other polymeric drug carriers is their ease of fabrication, facilitated by their inherent self-assembly properties. This feature has prompted extensive clinical investigations into various polymeric micelle formulations [81].

In one study, researchers developed PLGA-PEG-retinoic acid-based polymeric micelles to deliver the cancer drug irinotecan to HT-29 human colorectal and HepG2 cells. These smartly targeted nanomicelles exhibited greater cytotoxicity against HepG2 and HT-29 cells compared to non-targeted nanomicelles and free drugs [82]. In another research study, micelles of the PEG-poly(beta-amino ester) (PBAE)-PEG triblock copolymer were synthesized for the pH-dependent delivery of the water-insoluble cancer drug verteporfin [83].

Nano-immunotherapy holds promise for breast cancer treatment, yet patient responses vary, and cures remain elusive. To enhance efficacy, researchers investigate drugs that reprogram cancer-associated fibroblasts (CAFs) to improve therapy delivery and immune stimulation. Panagi M. et al. demonstrated the development of tranilast-loaded micelles, which achieve superior CAF reprogramming at a 100-fold lower dose compared to free drugs. Combining these micelles with epirubicin or Doxil and immunotherapy enhances T-cell infiltration, leading to cures and immunological memory in immunotherapy-resistant breast cancer in mice. Shear wave elastography (SWE) can monitor tumor stiffness changes induced by the micelles, serving as a potential biomarker for treatment response. This approach underscores micellar encapsulation as a promising strategy for reprogramming the tumor microenvironment, with SWE as a potential tool for treatment monitoring [84].

#### 2.2.3. Dendrimers

Dendrimers are a type of polymer often utilized in cancer nanomedicine. They are highly branched, tree-like molecules with well-defined structures and sizes, comprising three main components: a central core, branching units, and terminal groups. Drugs can be loaded onto dendrimers by encapsulating them in the core or binding them to the surface [85]. Dendrimers are biocompatible, easy to eliminate from the body, and can accumulate in tumors due to the EPR effect. However, cationic dendrimers can be toxic to normal cells due to their strong interaction with cell membranes, potential to disrupt cellular functions, induction of oxidative stress, triggering of immune responses, and size-related effects that can lead to cell damage and apoptosis [86]. Polyamidoamine dendrimers represent the most popular type of dendrimer employed in various biological applications, including drug delivery and imaging. They are commercially available in a wide range of generations and surface properties, rendering them highly versatile [87].

### 2.3. Nanocrystal

Drug nanocrystals offer a promising solution to the challenge of enhancing the bioavailability and water solubility of low-solubility drugs. They also feature a greater drug-loading capacity compared to nanocarrier drugs [88]. Furthermore, pure drug nanocrystals avoid the potential side effects associated with drug carriers. Therapies based on nanocrystals can be formulated into various dosage forms, including capsules, tablets, and pellets, enabling administration through different routes [89].

Over the past couple of decades, several nanocrystal products have received approval for use in diverse medical conditions. Rapamune, an immunosuppressant preventing organ rejection in kidney transplant patients, was the first nanocrystal drug to secure market approval [90]. Despite the approval of multiple nanocrystal drugs for clinical use, significant challenges to their widespread adoption still exist. One challenge arises from an incomplete understanding of the relationship between the structure and function of nanocrystal drugs. Another challenge emerges from the absence of standardized methods for characterizing nanoparticles. Consequently, the development of standards and regulatory policies for evaluating the safety and efficacy of nanocrystal drugs is considered essential [91]. Additionally, it is difficult to achieve a uniform dose [88]. Different ongoing research studies are focusing on the benefits of using nanocrystals in the field of cancer. For example, cellulose nanocrystals loaded with 5-fluorouracil, and salinomycin nanocrystals showed promising results in the treatment of colorectal cancer. Another study demonstrated the efficacy of albumin-coated carfilzomib nanocrystals in breast cancer therapy [92].

### 2.4. Nanoprotein

Protein nanoparticles play a crucial role in the development of targeted drug delivery for anticancer therapy due to their distinct properties. These protein nanoparticles are characterized by biodegradability, compatibility, and safety. Various proteins have been studied as potential nanoparticle vehicles for cancer therapy, including albumin, collagen, and gelatin, among others [93].

Abraxane (nab-paclitaxel) is a novel chemotherapy drug made with a protein called albumin. It is the first drug of its kind to receive Food and Drug Administration (FDA) approval. The traditional method of producing paclitaxel involved the use of solvents that could lead to side effects. However, Abraxane does not employ these solvents. Both preclinical and clinical studies have demonstrated that Abraxane is more effective in combating cancer cells and causes fewer side effects than the traditional method of paclitaxel production [94]. In 2008, Ontak (denileukin diftitox) gained approval for the treatment of non-Hodgkin’s peripheral T-cell lymphomas (PTCL). This formulation contains an interleukin (IL)-2 receptor antagonist, which is a cytotoxic targeting molecule, along with the nanoprotein [95].

### 2.5. Inorganic Nanoparticle

Inorganic nanocarriers are tiny particles made of various materials, including metals (gold and silver), metal oxides (iron oxide, titanium oxide, copper oxide, and zinc oxide), mesoporous silica, graphene oxide, carbon nanotubes, and black phosphorus [96,97]. They exhibit various shapes, such as nanoshells, nanorods, nanocages, nanostars, and nanospheres. The primary advantage of inorganic nanoparticles over organic nanocarriers is their high stability. For instance, inorganic nanocarriers exhibit greater stability than lipid-based nanocarriers, reducing the likelihood of degradation by oxygen or water. Additionally, drugs are less likely to leak from inorganic nanocarriers, making them a superior choice for drug delivery [98,99]. Table 2 demonstrates various inorganic nanomaterials and some of their applications [100,101,102,103,104,105].

### 2.6. Stealth Nanocarriers

Stealth nanocarriers are a critical component of nanomedicine, particularly in cancer treatment, due to their ability to enhance drug delivery efficiency [111]. These carriers are typically composed of biocompatible and biodegradable materials, such as lipids or polymers, and are coated with a hydrophilic polymer, most commonly PEG [111,112]. The PEG coating creates a stealth effect by reducing the recognition and clearance of the nanocarriers by the immune system, particularly by macrophages in the liver and spleen [61,113]. This stealth property prolongs the circulation time of the nanocarriers in the bloodstream, allowing for increased accumulation at the tumor site through the enhanced permeability and retention (EPR) effect. Additionally, stealth nanocarriers can improve the solubility and stability of encapsulated drugs, enhance their bioavailability, and reduce their toxicity [114]. Overall, stealth nanocarriers play a crucial role in improving the efficacy and safety of cancer therapeutics by optimizing drug delivery to tumor tissues while minimizing systemic side effects [115].

### 2.7. Comparison of Nanomaterial Platforms for Drug Delivery in Cancer Therapy

When comparing the advantages and disadvantages of different types of nanomaterial platforms for drug delivery, lipid-based nanoparticles, including liposomes, present several advantages. They are biocompatible, biodegradable, and can encapsulate both hydrophilic and hydrophobic drugs with a high loading capacity. They also enable controlled drug release and can be tailored for specific properties. However, conventional liposomes are quickly cleared from the bloodstream, limiting their effectiveness. This limitation has led to the development of stealth liposomes with extended circulation time. Additionally, although strategies like receptor targeting and stimuli-responsive drug release enhance their efficacy, another limitation is the possibility of leakage and fusion of encapsulated drug/molecules [60,116].

SLNs offer stability in aqueous environments, controlled drug release, and versatility in administration routes. They have shown promise in enhancing the efficacy of various anticancer drugs [42]. NLCs, the second generation of lipid nanoparticles, address limitations of SLNs, such as restricted drug loading and potential for drug leakage, by using a combination of solid and liquid lipids [117].

In comparison, polymer-based platforms, including polymer–drug conjugates, polymeric micelles, and dendrimers, offer ease of synthesis, high stability, controlled drug release, and low toxicity [118]. Polymer–drug conjugates can enhance pharmacokinetic properties and reduce side effects [119]. Polymeric micelles possess advantages over other polymeric drug carriers due to their inherent self-assembly properties, making them easier to fabricate [120]. Dendrimers, while versatile, can be toxic to normal cells due to their strong interaction with cell membranes [121].

Nanocrystals offer benefits such as enhanced bioavailability and water solubility of drugs, and high drug-loading capacity. However, challenges include an incomplete understanding of structure–function relationships, the difficulty in achieving an adjustable dosage range, and the lack of standardized characterization methods [88]. Inorganic nanoparticles, like gold and silver nanoparticles, offer high stability and reduced drug leakage, making them suitable for drug delivery. However, their potential toxicity to normal cells and complex synthesis processes are drawbacks [25,122]. Overall, each type of nanomaterial platform has its own set of advantages and limitations, and the choice of platform depends on the specific requirements of the drug delivery system.

## 3. Nanoparticle Properties and Characteristics

The development of a new nanomedication requires significant effort, especially when it is intended for complex diseases such as cancer, human immunodeficiency virus, and autoimmune diseases, or for targeting highly intricate sites such as the brain. A comprehensive understanding of nanoparticle characteristics is important for designing formulations tailored to specific therapeutic goals [123]. This section will discuss the key characteristics related to various nanoparticles and their contribution to the development of nanomedication for chemotherapy. These characteristics include physicochemical properties (size, shape, and charge), nanoparticle lipophilicity, and nanoparticle drug release.

### 3.1. Physiochemical Properties

#### 3.1.1. Nanoparticle Size

The standard size range of nanoparticles typically falls within the range of 10–1000 nm, with one dimension not exceeding 100 nm [23]. Additionally, nanoparticles can be characterized based on their volume-specific surface area (VSSA). Generally, particles possessing a VSSA equal to or exceeding 60 m^2^/cm^3^ of material volume are categorized as nanoparticles. Nanoparticles should not be too small, as this could allow them to be easily removed by the kidneys or to leak into capillaries. Consequently, they will have a short bioavailability, and a higher dose will be needed to target tumor cells. Conversely, they should not be too large, as this could make them susceptible to phagocytosis by the RES or clearance by the immune system. Considering all of these factors, the optimal diameter range for nanoparticles in the formulation of anticancer drugs is 10–150 nm [45,124].

The small size of nanoparticles plays a crucial role in improving the delivery of anticancer agents, which will be illustrated comprehensively in the drug delivery section. Furthermore, cellular uptake of drug molecules is greatly influenced by nanoparticle size. For example, studies have shown that nanospheres with a diameter of approximately 50 nm exhibit the highest cellular uptake when compared to particles of different sizes, as observed in the case of HeLa cells from carcinoma cell lines [125].

Furthermore, nanoparticles larger than 200 nm are quickly eliminated from the body compared to smaller nanoparticles, primarily due to their activation of the complement system. This underscores the critical role of nanoparticle size customization in achieving optimal drug delivery and bioavailability [126].

#### 3.1.2. The Shape of Nanoparticles

Nanoparticles come in a variety of shapes, including spheres, rods, and other complex geometries. Each shape imparts unique characteristics that impact their behavior. Nanoparticle shapes can be deliberately customized to enhance their effectiveness in tumor therapy [127,128]. For instance, oblate-shaped nanoparticles have shown the ability to deliver more antibodies to tumor cells compared to nanospheres of the same size and dosage. This effect is mainly attributed to the larger surface area of oblate nanoparticles, which enables them to bind with a greater number of antibodies [129].

In a study, trastuzumab-coated nanorods demonstrated a remarkable five-fold increase in inhibiting the growth of BT-474 breast cancer cells compared to equivalent nanospheres administered at the same nanoparticle dosage. This substantial improvement can be attributed to a 66% increase in binding and cellular uptake of the nanorods in contrast to the nanospheres [130].

Among the diverse shapes of nanocarriers, polymersomes stand out as self-assembled vesicles composed of amphiphilic block copolymers. Their unique structure mimics natural cell membranes, imparting excellent biocompatibility and stability. Polymersomes are hollow-shell structures, offering exceptional tunability in size, shape, and surface properties [131,132]. This makes them versatile platforms for drug encapsulation and delivery. They efficiently encapsulate both hydrophobic and hydrophilic drugs, broadening their therapeutic applications. Additionally, polymersomes exhibit a stealth-like behavior, evading immune recognition and prolonging circulation time in the bloodstream, which is advantageous for targeted drug delivery [132,133,134].

Various smart nanomedications have been developed using polymersomes for targeting tumor cells. For instance, Fathy F. et al. demonstrated the development of enzyme-loaded tumor-dilatable polymersomes for smart nanomedication in combination with chemo-immunotherapy. These polymersomes, initially 100 nm, swell to 200 nm in the acidic tumor microenvironment, allowing for prolonged retention in tumors. They enhance membrane permeability, enabling drug activation selectively at tumor sites. This approach effectively suppressed primary tumors with minimal systemic toxicity and inhibited distant tumors via antitumor immunity activation in mice [135].

Another study performed by Zhou Q. et al. utilized ferrocene-containing polymersome nanoreactors for combination with chemodynamic-immunotherapy. These nanoreactors co-loaded glucose oxidase (GOD) and the stimulator of interferon genes (STING) agonist symmetry-linked amidobenzimidazole (DiABZI), enhancing STING activation. The nanoreactors accumulated in tumor tissues, triggered by the acidic microenvironment, allowing for hydrogen peroxide (H_2_O_2_) production by GOD. This, along with the release of DiABZI, induced antitumor immune responses, effectively treating primary and metastatic tumors [136].

#### 3.1.3. Surface Charge of the Nanoparticle

The surface charge of nanoparticles should be carefully considered when developing new nanomedications, as it significantly influences formulation stability, distribution, and bioavailability [24,137]. In one study, it was reported that nanomedications with a positive surface charge showed remarkable results in targeting tumor vessels. However, after penetrating the tumor cells, a shift from a positive to a neutral surface charge enhances distribution within the tumor tissue [29].

Nanoparticle charge can also enhance selectivity for the target site. For instance, a strong electrostatic attraction between ZnO nanoparticles and cancer cells promotes uptake and drug accumulation in the target tissue. This phenomenon can be explained by the fact that under physiological conditions, such as blood or tissue fluid with a pH of seven, ZnO nanoparticles carry a positive charge, while cancer cells typically exhibit a high concentration of negatively charged anionic phospholipids on their outer membrane [138,139].

### 3.2. Nanoparticle Lipophilicity

The lipophilicity of nanoparticles can significantly impact the pharmacokinetic properties of a formulation. Nanomedications with high lipophilicity tend to be eliminated more rapidly from the bloodstream due to the action of the RES [45]. The RES efficiently removes lipophilic particles from circulation, typically directing them to the spleen or liver for clearance. Consequently, this rapid elimination can reduce the accumulation of the cancer-therapeutical agent within the tumor tissue [140]. To address this limitation, researchers have developed various techniques, as discussed in the previous section. These techniques include the use of PEGylated nanoparticles, which involve attaching PEG to the surface of nanoparticles to enhance their circulation time and reduce RES clearance [141]. Additionally, different ligand types, such as antibodies, aptamers, and others, have been explored to enhance the targeting and retention of nanoparticles within the tumor tissue [142].

### 3.3. Nanoparticle Drug Release

The primary goal of using nanoparticles extends beyond drug delivery to the target site; it also involves considering the release of the medication. Several elements can affect drug release, including formulation composition, chemical bonding, drug dispersion within the formulation, drug solubility, and the pH of the targeted site [29,143,144]. For example, polymeric nanoparticles can be categorized into two types, namely nanocapsules and nanospheres; each one has a different release mechanism. Nanocapsules contain a drug reservoir within the polymer structure, resembling vesicles. Nanospheres constitute a homogeneous system in which polymer chains form a matrix, evenly dispersing the drug throughout [124,145].

In nanospheres, drug release primarily occurs through matrix erosion, where the matrix degrades over time, releasing the drug. The initial drug release is often rapid due to the presence of weakly bound drug molecules on the surface of the nanoparticle, followed by sustained release over time. In the case of nanocapsules, drug release is controlled by the diffusion of the drug through the polymer layer. The rate of drug release depends on the diffusivity of the drug through the polymer layer, which is influenced by the type of polymer used and the size of the drug molecules [143,146].

## 4. Nanoparticle-Based Cancer Drug Delivery Systems

Currently, researchers and scientists are focused on improving the delivery of nanomedications for cancer therapy. As mentioned in the nanomaterial section, there are numerous examples of nanoformulations developed to deliver drugs to the target tumor site using different strategies, such as passive, active, or stimulus-responsive delivery [115,147]. Lately, smart targeted delivery systems have emerged as promising candidates for cancer therapy due to their high efficacy and minimal side effects, although most of these formulations are still in the clinical trial phase [148]. This section will focus on passive targeting and active targeting, as well as exogenous and endogenous stimulus-driven systems, in addition to smart targeted drug delivery systems.

### 4.1. Smart Targeted Therapy

A smart targeted nanoparticle carrier system is composed of three primary components: the nanoparticle responsible for transporting the anticancer agent, a selective mechanism designed to deliver the carrier system precisely to the target site, and a stimuli technique for controlled drug release at a specified rate and extent [110,149]. Using nanoparticles solely as carriers for drugs without considering these factors results in a conventional nanoparticle carrier system, lacking the smart targeting aspect. Such systems cannot exclusively release medication to the target site in the desired amount in response to external or internal stimuli [150,151].

Smart nanoparticles exhibit distinct characteristics that set them apart from conventional drug delivery systems. These nanoparticles can evade the immune system through PEGylation, where the nanoparticle surface is coated with PEG to prevent recognition and clearance, enabling longer circulation times [152]. They are also designed for targeted accumulation in specific tissues, such as tumors, achieved by surface modifications with ligands that bind to overexpressed proteins on target cells, enhancing drug delivery specificity while reducing off-target effects [153]. Additionally, smart nanoparticles enable controlled drug delivery, releasing therapeutic agents at precise locations and concentrations in response to external or internal stimuli. This control is often achieved by modifying the nanoparticle surface with various chemical groups to modulate drug release [153]. Furthermore, smart nanoparticles can co-deliver multiple substances, including anti-cancer drugs, genetic materials, and imaging agents, which is particularly beneficial in cancer therapy for enhancing treatment effectiveness. Through these capabilities, smart nanoparticles offer promising prospects for advancing cancer treatment strategies [154].

Table 3 provides an overview of the distinct characteristics of smart nanoparticles and some strategies for achieving a smart targeted nanoparticle delivery system of anticancer agents [152,153]. Most of the mentioned strategies have been previously used in developing active drug delivery or targeting TME. However, smart targeted drug delivery employs multiple strategies to achieve high efficacy. Formulating this type of carrier is challenging and complex, which explains their slow progress.

Other features currently undergoing extensive research hold significant potential for advancing the field of smart nanomedication. For example, transcytosable nanomedicine and immuno-oncological nanomedicine are two areas of particular interest [155].

Transcytosis is a crucial transport mechanism involving the vesicular movement of large molecules across epithelial or endothelial barriers, playing a vital role in maintaining tissue metabolism and homeostasis. While much attention has been given to paracellular transport, researchers have also explored the active transcellular pathway of transcytosis. Nanomedicine leverages various forms of transcytosis for efficient transport across tumor and brain endothelium, including receptor-mediated transcytosis (RMT), absorptive-mediated transcytosis (AMT), and bulk-phase or fluid-phase transcytosis (FPT) [156].

A study conducted by Wang et al. introduces a novel approach for treating triple-negative breast cancer (TNBC) using a paclitaxel (PTX) conjugate named PTX-SM-TAR. This conjugate, based on the peptide–drug conjugates (PDCs) strategy, combines a tumor-targeting peptide (A7R) and a cell-penetrating peptide (TAT) to modify PTX. PTX-SM-TAR self-assembles into nanoparticles, improving PTX’s water solubility. The nanoparticles are transcytosable, targeting receptors and mediating endocytosis by binding to neuropilin-1 (NRP-1). In vivo studies demonstrate that PTX-SM-TAR nanoparticles exhibit superior antitumor effects compared to PTX alone, offering a promising targeted delivery system for PTX in TNBC treatment [157].

Immunooncological nanomedicine integrates nanotechnology with immunotherapy, aiming to deliver immunomodulatory agents directly to tumors. This approach enhances treatment efficacy while minimizing systemic toxicity. By encapsulating agents such as immune checkpoint inhibitors or cytokines, these nanocarriers modulate the tumor microenvironment, overcoming immune evasion mechanisms and stimulating antitumor immune responses [158]. For instance, the use of cyclodextrin nanoparticles containing the Toll-like receptor 7/8 (TLR7/8) agonist R848 has demonstrated improved outcomes in cancer immunotherapy. This approach facilitates the transformation of tumor-associated macrophages (TAMs) into an M1-like phenotype, thereby enhancing the body’s immune response against cancer cells [159].

### 4.2. Passive Drug Delivery

Tumors cause the surrounding blood vessels to become less rigid and more permeable. This allows drug-loaded nanoparticles to accumulate in the tumor at a much higher rate than in normal tissue. This phenomenon is known as the enhanced permeability effect. Additionally, the lymphatic system in tumors is often poorly developed, which prevents nanoparticles from being drained away as quickly as they would in normal tissue. This is known as the enhanced retention effect. Together, these two effects are referred to as the EPR effect [160,161].

Although passive targeting represents a significant improvement over conventional chemotherapy, several obstacles may hinder the efficacy of this targeting system. For example, the EPR effect tends to be lower in tumors as the interstitial fluid pressure increases. Moreover, the EPR effect can vary significantly within and between tumors, even within the same patient [161,162]. Additionally, nanoparticles may be cleared by the RES, which consists of macrophages located in organs such as the liver and spleen. These macrophages are responsible for recognizing and clearing foreign particles, including nanoparticles, from the bloodstream. This poses a challenge for drug delivery because it can result in the rapid clearance of nanoparticles from circulation [160,163]. Therefore, various modifications to conventional nanoparticle vehicle systems may be applied to enhance the delivery of anticancer drugs to the targeted site.

### 4.3. Active Drug Delivery

Active targeting (ligand-based active tumor-targeting) is a strategy for selectively guiding drug-loaded nanoparticles to cancer cells. Cancer cells often display amplified or overexpressed cell surface receptors, also known as cell markers [142,164]. Drug-loaded nanoparticles can be conjugated with targeting ligands, which are molecules that bind to specific cell surface receptors. When the nanoparticles bind to these cell surface receptors, they are internalized into the cancer cells through receptor-mediated endocytosis, thereby maximizing drug accumulation [164,165]. Table 4 demonstrates a diverse range of receptors that are overexpressed on different cancer cells, along with the corresponding targeting moieties that can bind to these receptors and a brief description [47,165,166,167,168].

### 4.4. Tumor Microenvironment (TME) Responsive Drug Delivery

The next generation of nanoparticles for delivering anticancer agents represents a significant advancement in targeted smart drug delivery. This innovative approach focuses on selectively releasing drugs at specific tumor sites, harnessing the distinct microenvironmental characteristics of the tumor cells when compared to healthy ones [148,170]. Consequently, scientists formulate a smart nanoparticle delivery system to precisely release anticancer drugs within this specialized environment. There are two types of TME-responsive drug delivery: endogenous stimulus (intrinsic stimulus) systems and extrinsic stimulus systems [148,171].

The intrinsic stimulus emerges from differences in pH levels between normal cells and tumor cells, along with enzyme conversions, temperature discrepancies, and redox reactions. These unique features of the TME trigger the controlled release of therapeutic agents, ensuring the precise release of the medication where needed. Table 5 demonstrates a summary of various endogenous stimulus factors with some recent formulations [172,173,174,175,176].

Extrinsic stimulus systems utilize external factors to control the release of drugs from nanocarriers delivered to tumor sites. External factors such as a magnetic field, ultrasound waves, light, or electric fields activate the accumulated drug-loaded nanocarriers, prompting them to release drugs at the optimal concentration. This approach significantly enhances drug delivery precision and efficacy. Table 6 presents a summary of various exogenous stimulus factors with some recent formulations [179,180,181,182].

## 5. Contemporary Landscape in the Field of Cancer Nanomedicine

Current cancer treatments primarily encompass surgical procedures, radiation therapy, and chemotherapy. However, these three methods come with certain limitations, such as the inability to entirely eradicate cancerous cells and the potential harm to healthy tissue. The field of nanotechnology offers precise solutions that enable the targeted delivery of chemotherapy to cancer cells, provide guidance for surgical tumor removal, and enhance the effectiveness of radiation-based and other established therapies. These advancements hold the promise of reducing patient risks and improving overall survival rates [72,161,186]. A diverse range of nanoparticle-based pharmaceuticals has entered the global market for cancer therapy, as shown in Table 7. The availability and utilization of such products from numerous worldwide companies illustrate the success of nanomaterials as carrier agents for different chemotherapy treatments [27,37,110,150,187,188,189].

## 6. Challenges Encountered in Developing Nanomedication

Over the past couple of decades, the understanding of various physicochemical properties, characteristics, and different methods of preparation has significantly increased due to extensive research efforts. However, the field of nanomedication is still in the developmental stage due to the variety of challenges encountered throughout the development process. These obstacles have slowed down the introduction of new nanomedications for cancer into clinical usage [24,189,204]. This section discusses these challenges and presents some valuable solutions.

### 6.1. Overcoming EPR-Based Limitations

One of these challenges is the limitation encountered by the first generation of nanomedication. Most cancer nanomedicines available on the market rely on the EPR effect for passive tumor targeting. However, the EPR effect is influenced by the heterogeneity of tumors, resulting in variations between different patients and even within the same patient. Additionally, some evidence suggests that the EPR effect is more prominent in smaller animal models compared to humans, potentially leading to biased data regarding the effectiveness of a specific treatment [80,152]. To address the challenges posed by first-generation nanomedicines, researchers have developed advanced nanocarriers designed to enhance their ability to target tumors and achieve stimuli-responsive drug release, thereby creating smart nano medications capable of overcoming this challenge [21,205].

### 6.2. Ensuring Nanomaterial Safety and Effectiveness

Another challenge arises from the uncertainty surrounding the safety of the utilized nanomaterials. Despite numerous clinical studies conducted on this topic, the majority of them have focused on bulk materials. Since nanomaterials possess distinct physicochemical properties, these studies may not accurately reflect the true safety profile of nanomaterials. Furthermore, it is essential to conduct prolonged studies to assess the safety and biodegradability of nanomaterials over an extended period [206,207].

To proactively address potential toxicological reactions to emerging nanomaterials, a comprehensive understanding of their absorption, distribution, metabolism, and excretion in humans is crucial. Moreover, any modifications to the synthesis process, reagent selection, manufacturing techniques, or delivery methods may introduce variations in toxicity, necessitating the need for up-to-date safety assessments [56,208]. The clinical implementation of nanotherapeutics is fraught with significant concerns related to safety and quality assurance. Notably, even after receiving clinical authorization, a nanotherapeutic might be withdrawn from the market due to safety concerns [189,209].

Another significant challenge in utilizing nanomaterials in terms of safety and efficacy for cancer therapy is selecting suitable models. Current research predominantly relies on cell and animal models, which may not provide an accurate assessment of the anticancer efficacy of diagnostic and therapeutic agents. These models, subjected to various chemical and physical stresses, may fail to represent the conditions of entire human organs accurately. Replicating a reaction within the complex human system using a single model poses considerable difficulties [147,210].

Nevertheless, integrating multiple models capable of simulating in vivo interactions, the extracellular matrix, intercellular signaling, and growth can offer a system that more closely aligns with and enhances our understanding of in vivo events. Biomimetic organ/tumor-on-a-chip tools [211] and three-dimensional cell culture model systems are promising strategies to replicate the in vivo conditions experienced by nanocarriers in cancer patients [212].

### 6.3. Scaling up Nanoparticle Production

The production of a large quantity of nanomedications is one of the most complicated challenges for several reasons. The high cost associated with scaling up nanotherapies is driven by increasing research and manufacturing expenses, as well as clinical trial expenditures [213]. Various laboratory techniques, such as nanoprecipitation, ionic gelation, sonication, and supercritical fluid technology, are used to synthesize nanocarriers. These methods require close monitoring of formulation parameters for reproducibility, with some demonstrating satisfactory scale-up potential. However, applying them to large-scale multifunctional nanocarriers remains uncertain, especially for those with surface modifications or TME responsiveness [214,215]. Therefore, it is essential to devise an affordable synthesis technique and a straightforward purification process to facilitate the large-scale production of nanomaterials [216].

### 6.4. Regulatory Hurdles in Nanotherapeutics Development

The lack of regulation and standards in manufacturing practices, quality control, safety, and efficacy assessment present barriers to the development of nanotherapeutics. Currently, there are no international regulatory standards established for the clinical translation of nanotherapeutics. Regulatory bodies, such as the FDA and EMA, have only formulated draft guidelines [217]. A compelling example of this phenomenon is the fact that nanotherapeutics approved in one country may not be approved in another, illustrating significant regional differences in approaches to nanotechnology applications [218]. To overcome this challenge, enhanced collaboration and communication among researchers, regulatory bodies, and industry stakeholders are crucial for developing precise guidelines and standards for the characterization and evaluation of nanocarriers [115].

### 6.5. Ethical Considerations

The ethical considerations regarding nanotechnology in cancer therapy are complex and require meticulous attention. Ensuring patient safety is crucial for any medical intervention, including nanotechnology-based treatments. The distinct properties of nanomaterials necessitate a thorough examination of potential unexpected side effects or long-term implications prior to patient application [115]. Rigorous and transparent preclinical testing, including extensive toxicity studies and a detailed understanding of nanomaterial interactions with biological systems, is essential [219].

Informed consent is crucial in nanotechnology for cancer therapy, ensuring patients in clinical trials are fully aware of the treatment’s experimental nature, potential risks, and efficacy uncertainties. The complexity of nanotherapies demands clear patient understanding for informed decision-making. The consent process should be transparent, respectful, and tailored to the patient’s understanding, enabling active participation in healthcare decisions. Additionally, the broader societal impact of nanotechnology-based cancer therapies requires ethical consideration [115].

## 7. Conclusions and Future Perspective

Overall, nanomaterials have introduced a diverse range of carrier systems for chemotherapy in cancer treatment, each possessing unique characteristics. Significant progress in this area has led to innovative delivery strategies that enhance drug selectivity and reduce the side effects associated with conventional chemotherapy. Despite considerable efforts and resources invested, challenges continue to persist, impeding progress in cancer research and nanomedicine. While most smart targeted nanomedications remain in research or clinical trial stages, they exhibit promising potential for cancer therapy.

In future perspectives, researchers are advancing the design and functionality of nanocarriers, focusing on improving their stability in the bloodstream, enhancing drug-loading capacities, and refining targeting abilities. Advances in materials science and nanofabrication techniques enable the creation of nanocarriers with precise properties, such as size, shape, and surface chemistry, affecting their body interactions. The development of smart nanocarriers is also gaining momentum, allowing for simultaneous diagnosis, monitoring, and treatment of cancer, leading to more personalized and precise therapy [115,220].

Future directions in nanotechnology for cancer therapy should also investigate the possibilities offered by immunotherapeutic strategies. Immunotherapy has transformed cancer treatment by utilizing the body’s immune system to attack cancer cells. Combining nanotechnology with immunotherapy can create more powerful and targeted cancer therapies. Nanoparticles can be engineered to deliver immune-boosting molecules, like checkpoint inhibitors or cytokines, directly to the tumor, reducing off-target effects and enhancing the immune response against cancer cells, thus improving therapeutic outcomes [115,221].

Additionally, the advancement of personalized nanomedicine represents a promising avenue in the battle against cancer. With the expanding knowledge of cancer’s genetic and molecular foundations, nanotechnology can be harnessed to develop therapies tailored to individual patients. By customizing nanocarriers to match the distinct genetic makeup of a patient’s tumor, drug delivery and treatment efficacy can be optimized [218,222].

A critical aspect of the future of nanotechnology in cancer therapy is the transition of laboratory discoveries into clinical practice. Overcoming the hurdle between benchtop research and clinical applications is a vital challenge. As the field progresses, bridging this gap through collaborative efforts between researchers, clinicians, and regulatory agencies will be essential for the successful translation of nanotechnologies into effective cancer treatments [223,224].

## Figures and Tables

**Table 1:** Applications of Lipid-Based Nanoparticles in Cancer Therapy.

Lipid-Based Nanoparticle	Examples of Prominent Applications	Reference
Liposomes	Attachment of anti-CD22 monoclonal antibodies to PEGylated liposomes loaded with doxorubicin (DOX) for enhanced drug accumulation in non-Hodgkin’s lymphoma tumors.	[44]
	Evaluation of paclitaxel liposomes targeting the folate receptor (FR) in cancer therapy.	[20]
	Evaluation of TfR-targeted liposomes (Tf-PEG-liposomes) in a mouse model of colon cancer.	[45]
	Investigation of liposomes functionalized with the neurofilament-derived peptide, NFL-TBS.40–63, for targeted delivery to glioblastoma cells across the blood–brain barrier (BBB).	[46]
	Phase I trial of siRNA targeting EPHA2 with liposomes (siRNA-EPHA2-DOPC) for advanced neoplasm.	[47]
	Phase III trial of liposomal paclitaxel (EndoTAG-1) for breast cancer.	[48]
	Phase III trial of HER2-targeted liposomal doxorubicin hydrochloride (MM-302) for breast cancer.	[49]
	Phase III trials of thermally sensitive liposomal doxorubicin (ThermoDox) for breast cancer.	[15]
Solid Lipid Nanoparticles	Evaluation of solid lipid nanoparticles (SLNs) loaded with gemcitabine on patient-derived primary pancreatic cancer cell lines (PPCL-46) and MiaPaCa-2 pancreatic cancer cell lines.	[50]
	Development of transferrin-conjugated solid lipid nanoparticles (SLNs) for targeted delivery of tamoxifen citrate for breast cancer treatment.	[51]
	Development of arginine-glycine-aspartic (RGD) tripeptide-modified solid lipid nanoparticles (SLNs) for targeted delivery of doxorubicin (DOX) for breast cancer treatment.	[52]
Nanostructured Lipid Carriers	Evaluation of folate-chitosan-coated nanostructured lipid carriers (FCH-NLCs) encapsulating harmaline for targeted breast cancer therapy.	[53]
	Optimization of nanostructured lipid carriers (NLCs) loaded with metformin and thymoquinone for breast cancer therapy.	[54]

**Table 2:** Inorganic Nanoparticles and Their Prominent Applications.

Type of the Inorganic Nanoparticle	Characteristics	Examples of Prominent Applications	Reference
Gold Nanoparticles (AuNPs)	AuNPs have unique thermal and optical properties, which can be controlled by changing its size, shape, and/or surface chemistry.	Chitosan-folic acid-coated gold nanoparticles are biocompatible and can be used to deliver drugs more selectively to tumor cells.	[106]
Silver Nanoparticles (AgNPs)	AgNPs, typically smaller than 100 nm and composed of 20 to 15,000 silver atoms, have unique physicochemical and biological properties that are influenced by their size and shape.	C225-coated Ag NPs (Ag/C225) are effective radiosensitizers for nasopharyngeal carcinoma epithelial cell lines, with an average preserved anti-EGFR antibody activity of about 82%.	[107]
Iron Oxide Nanoparticles	Iron oxide nanoparticles are made up of a solid iron oxide core surrounded by a layer of water-soluble polymers such as dextran or sucrose.	A nanotherm is a type of nanoparticle made up of iron oxide coated with aminosilane. It is used to eradicate cancer cells by heating them with an alternating magnetic field.	[108]
Copper Nanoparticles (CuNPs)	CuNPs exhibit strong near-infrared light absorption and can generate heat. This characteristic makes them valuable in photothermal therapy.	Gold and copper nanoparticles have the potential to be used in the treatment of breast cancer when applied to MCF-7 and MDA-MB-231 breast cancer cells.	[103]
Titanium Dioxide Nanoparticles (TiO_2_ NPs)	TiO_2_ NPs can serve as anti-cancer agents due to their significant cellular accumulation, which can induce alterations in metabolic pathways, ultimately resulting in necrosis.	TiO_2_ has been used to deliver various anti-cancer drugs, including daunorubicin, temozolomide, doxorubicin, and cisplatin, to cancer cells.	[109]
Mesoporous Silica Nanoparticles (MSNs)	MSNs can transform crystalline drugs into their amorphous state, facilitating enhanced cellular absorption.	MSNs increased paclitaxel cytotoxicity by 4.3-fold against HepG2 cells and camptothecin cytotoxicity by 86% against Capan-1 human pancreatic adenocarcinoma cells.	[110]

**Table 3:** Smart Nanoparticle Characteristics and Strategies.

Characteristics of Smart Nanoparticles	Strategies for Smart Nanoparticles	Reference
Immune System Evasion	Achieved through PEGylation to evade immune system clearance.	[152]
Targeted Accumulation	Surface modification with ligands matching cancer cell overexpressed proteins for precise targeting.	[153]
Controlled Delivery	Delivering therapeutic agents to the desired location at specific concentrations, using external or internal stimuli. Achieving this control often involves grafting various chemical groups onto the surface of the nanocarrier.	[153]
Co-Delivery Capability	Capable of delivering multiple substances, such as anti-cancer drugs, genetic materials, and imaging agents.	[154]

**Table 4:** Different Targeting Ligands for Overexpressed Receptors in Tumor Cells.

Receptor	Targeting Ligands	Description	Reference
Folate receptor (FR)	Folic acid (FA)	FA, also known as vitamin B9, is crucial for DNA-related processes. When combined with the FR, it enters cancer cells through endocytosis. FR is highly expressed in various epithelial tumors, including ovarian, lung, breast, endometrial, cervical, renal, bladder, and brain cancers.	[165]
Integrin receptor	Arginylglycylaspartic acid peptide (RGD)	The RGD peptide is a common integrin-binding moiety found in the extracellular matrix. It binds most strongly to αvβ3 and αvβ5 integrins, which are not expressed in normal tissues. Mainly in lung cancer and breast cancer.	[166]
Epidermal Factor Receptor (EGFR)	Anti-EGFR	EGFR is a transmembrane glycoprotein in the tyrosine kinase receptor family. EGFR plays a huge role in the development of several cancers, such as colon, non-small-cell lung, breast, head, and ovarian cancers.	[47]
Transferrin receptor (TfR)	Transferrinreceptor ligand	TfR regulates iron distribution in normal human cells. TfR is more expressed in breast cancer, glioma, lung adenocarcinoma, and chronic lymphocytic leukemia.	[167]
Cluster of Differentiation 44 (CD44)	Hyaluronic acid	CD44, a transmembrane adhesion glycoprotein, participates in various physiological and pathological pathways, especially in tumor development, progression, and metastasis. CD44 is overexpressed on the surfaces of many tumors, including liver, breast, colon, and lymphoma.	[169]

**Table 5:** Endogenous Stimulus Factors with Corresponding Examples.

Endogenous Stimulus Factor	Description	Example	Reference
The pH-responsive stimulus	The variance in pH levels between normal and cancer cells provides a robust basis for developing a stimulus-responsive drug delivery system.	A mesoporous silica nanoparticle-chitosan system was prepared for pH-responsive drug delivery, demonstrating enhanced Ibuprofen release at pH 6.8 over pH 7.4, promising for breast cancer treatment.	[122]
Redox sensitive stimulus	Glutathione sulfhydryl (GSH), a potent antioxidant, is abundant in mammalian tissues, especially within tumors, where its concentration is four times higher than in normal cells. GSH can reduce disulfide bonds in nanocarriers, leading to precise drug release, making it a key component in targeted drug delivery.	Stable micelles were developed by coupling heparosan with deoxycholic acid via disulfide bonds to deliver DOX to cancer tissues. These micelles exhibited strong drug-loading capacity and glutathione-triggered drug release.	[177,178]
Enzyme stimulus	Extracellular enzymes target tumor sites due to elevated activity but are not suitable for intracellular drug release because enzyme levels in cancer and healthy cells are similar. Proteases are ideal for drug release from liposomes.	Doxorubicin-loaded GLFG liposomes, degraded by overexpressed cathepsin B in cancer cells, effectively inhibited cancer cell proliferation in Hep G2 cells.	[175]

**Table 6:** Exogenous Stimulus Factors with Corresponding Examples.

Exogenous Stimulus Factor	Description	Example	Reference
Magnetic field responsive stimulus	Magnetic systems attract drug-loaded nanocarriers to tumor sites using an extracorporeal magnetic field.	Implantable magnetic chitosan hydrogel loaded with both rifampicin and adriamycin drugs responds to low-frequency alternating magnetic fields, releasing drugs intermittently without inducing magnetic hyperthermia, enhancing precision and reducing post-surgical infection risk.	[183]
Thermo-responsive stimulus	Exceeding the critical solution temperature of the polymer nanoparticle disrupts the hydrophilic–hydrophobic balance, leading to polymer chain dehydration and structural changes, releasing the drug.	Superparamagnetic nanoparticles loaded with camptothecin and formulated to be thermo-responsive. This nanocomposite enhanced cytotoxicity against cancer cells compared to free drugs.	[184]
Light triggered stimulus	Light-responsive drug delivery systems achieve precise drug release upon exposure to external light sources, including visible, infrared, or ultraviolet light.	The release of DOX from the gold nanocarrier is enhanced when exposed to 808 nm illumination.	[181]
Ultrasound responsive stimulus	It can induce both mechanical and thermal effects within nanocarriers, leading to the release of loaded medications.	Ultrasound-sensitive nanobubbles loaded with paclitaxel and siRNA for hepatocellular carcinoma were developed. When exposed to low-frequency ultrasound, this system induces apoptosis in cancer cells and reduces tumor volume.	[185]

**Table 7:** Approved Nanomedication Products for Various Cancer Treatments and Their Utilized Nanomaterials.

Product (Active Ingredient)	Type of Nanomaterial	Indication(s)	Developer	Initial Approved Year and Region	Reference
Doxil (Doxorubicin)	PEGylated liposome	Kaposi’s sarcoma, breast cancer, ovarian cancer, multiple myeloma	Janssen	FDA (1995) EMA (1996)	[49]
DaunoXome (Daunorubicin)	Liposome	Kaposi’s sarcoma	Galen	FDA (1996)	[190]
Lipo-Dox (Doxorubicin)	PEGylated liposome	Kaposi’s sarcoma, breast cancer, ovarian cancer	Taiwan Liposome	Taiwan (1998)	[33]
DepoCyt (Cytarabine)	Liposome	Lymphomatous meningitis	Pacira Pharmaceuticals	FDA (1999)	[191]
Myocet (Doxorubicin)	Liposome	Metastatic breast cancer	Teva UK	EMA (2000)	[192]
Eligard (Leuprolide acetate)	Polymer	Prostate cancer	Tolmar Pharmaceuticals	FDA (2002)	[193]
Zevalin (90Y-ibritumomab tiuxetan)	Liposome	Lymphoma	Bayer Pharma	FDA (2002) EMA (2004)	[194]
Abraxane (Paclitaxel)	Albumin nanoparticle	Advanced NSCLC, metastatic breast cancer, metastatic pancreatic cancer	Abraxis BioScience/Celgene	FDA (2005) EMA (2008)	[127]
Oncaspar (L-asparaginase)	Polymer protein conjugate	NSCLC, ovarian cancer, and breast cancer	Les Laboratoires Servier	State Food and Drug Administration of China (2006)	[127]
Genexol-PM (Paclitaxel)	PEG-b-PLA polymeric micelle	Breast cancer, ovarian cancer, and NSCLC	Samyang Biopharmaceutical	South Korea (2007)	[80]
Mepact (Mifamurtide)	Liposome	Osteosarcoma	Takeda	EMA (2009)	[195]
NanoTherm	Iron oxide nanoparticle	Thermal ablation of glioblastoma, prostate cancer	MagForce Nano	EMA (2010) FDA (2018)	[196]
Marqibo (Vincristine)	Liposome	Acute lymphoblastic leukemia	Talon Therapeutics Inc.	FDA (2012)	[197]
Opaxio (Paclitaxel)	Polymer	Head and neck cancer; Glioblastoma	Cell Therapeutics, Inc.	FDA (2012)	[198]
Ryanodex (Dantrolene sodium)	Nanocrystal	Malignant hypothermia	Eagle Pharmaceuticals	FDA (2014)	[199]
Onivyde (Irinotecan)	PEGylated liposome	Metastatic pancreatic cancer	Merrimack Pharmaceuticals	FDA (2015)	[200]
DHP107 (Paclitaxel)	Lipid nanoparticle	Gastric cancer	Daehwa Pharmaceutical	South Korea (2016)	[201]
Vyxeos CPX-351 (Daunorubicin:cytarabine [1:5 molar ratio])	Liposome	Acute myeloid leukemia	Jazz Pharmaceuticals	FDA (2017) EMA (2018)	[202]
Apealea (Paclitaxel)	Micelle	Ovarian, peritoneal, and fallopian tube cancer	Oasmia Pharmaceutical	EMA (2018)	[28]
Hensify	Hafnium oxide nanoparticle	Locally advanced soft tissue sarcoma	Nanobiotix	CE mark (2019)	[203]

CE mark: European market approval; EMA: European Medicines Agency; FDA: U.S. Food and Drug Administration; NSCLC: non-small-cell lung cancer.

## Data Availability

No data is associated with this article.

## Appendix A

**Table A1:** FDA-Approved Nanomedications: Brand Names, Developers, Indications [27,37,110,150,187,188,189,225].

Brand Name (Active Ingredient)	Developer	Indication(s) [Approval Year and Region]
Polymer Nanoparticle		
Adagen^®^ (Pegademase bovine)	Sigma-Tau Pharmaceuticals	Adensine deaminase (ADA) deficiency in patients with severe combined immunodeficiency disease (SCID) [1990 US] Disc. * 2019
Adynovate^®^ (Antihemophilic factor) (recombinant), PEGylated	Shire	Hemophilia [2015 US]
Cimzia^®^ (Certolizumab pegol)	UCB	Crohn’s disease [2008 US] [2009 EU]
Copaxone^®^; Glatopa ^®^ (Glatiramer acetate)	Teva Neuroscience	Multiple sclerosis (MS) [1996 US]
Apealea^®^ (paclitaxel)	Oasmia Pharmaceutical AB	Ovarian cancer, peritoneal cancer, fallopian tube cancer [2018 EU]
Genexol-PM^®^ (paclitaxel)	Lupin Ltd.	Breast cancer [2007 US]
Eligard^®^ (Leuprolide acetate)	Tolmar Pharmaceuticals	Prostate cancer [2002 US]
Krystexxa^®^ (Pegloticase)	Crealta Pharmaceuticals	Chronic gout [2010 US] [2013 EU]
Macugen^®^ (Pegaptanib)	Bausch & Lomb	Neovascular age-related macular degeneration [2004 US] [2006 EU] Disc. *
Mircera^®^ (mPEG-epoetin beta)	Roche	Anemia associated with chronic kidney disease [2007 US/EU]
Neulasta^®^ (Pegfilgrastim)	Amgen	Chemotherapy induced neutropenia [2002 US/EU]
Oncaspar^®^ (Pegaspargase)	Shire	Acute lymphoblastic leukemia [1994 US] [2016 EU]
PegIntron^®^ (alpha interferon (INF) molecule)	Merk & Co. Inc.	Hepatitis C [2000 EU] [2001 US]
Pegasys^®^ (Peginterferon alpha-2a)	Roche	Hepatitis B and C [2002 US/EU]
Plegridy^®^ (Peginterferon beta-1a)	Biogen	Multiple sclerosis (MS) [2014 US/EU]
Renvela^®^/Renagel^®^ (Sevelamer hydrochloride)	Genzyme	Chronic kidney disease [2000 US/EU]
Mircera (epoetin β (EPO)	Vifor	Anemia [2007 EU] [2018 US]
Macugen^®^ (pegatinib sodium)	Pfizer	Choroidal neovascularization caused by wet age-related macular degeneration [2004 US]
Somavert^®^ (Pegvisomant)	Pfizer	Acromegaly [2003 US] [2002 EU]
Rebinyn^®^ (recombinant DNA-derived coagulation FIX)	NovoNordisk	Hemophilia B [2017 US]
Restasis^®^ (cyclosporine)	Allergan	Chronic dry eye [2003 US]
Zilretta^®^ (Triamcinolone acetonide)	Flexion Therapeutics	Osteoarthritis knee pain [2017 US]
Sublocade^®^ (Buprenorphine)	Indivior	Opioid use disorder [2017 US]
Liposome Nanoparticle		
Abelcet^®^ (Amphotericin B)	Sigma-Tau Pharmaceuticals	Fungal infections [1995 US]
AmBisome^®^ (Amphotericin B)	Gilead Sciences	Fungal/protozoal infections [1997 US]
Curosurf^®^ (Poractant alpha)	Chiesei	Respiratory distress syndrome [1999 US]
DaunoXome^®^ (Daunorubicin)	Galen	Kaposi’s sarcoma [1996 US] Disc. * 2016
DepoCyt© (Cytarabine)	Pacira Pharmaceuticals	Lymphomatous meningitis [1999 US] [2001 EU] Disc. * 2017
Zevalin^®^ (90Y-ibritumomab tiuxetan)	Bayer Pharma	lymphoma [2002 US] [2004 EU]
DepoDur^®^ (Morphine sulfate)	Pacira Pharmaceuticals	Analgesia (post-operative) [2004 US] Disc. *
Doxil^®^/CaelyxTM (Doxorubicin)	Janssen	Karposi’s sarcoma [1995 US] [1996 EU] Ovarian cancer [2005 US] [1996 EU] Multiple myeloma [2008 US] [1996 EU] Breast cancer [1996 EU]
Lipodox^®^ (doxorubicin)	doxorubicinSun Pharma Global FZE	Metastatic ovarian cancer, HIV-associated KS [2013 US]
Marqibo^®^ (Vincristine sulfate)	Spectrum Pharmaceuticals	Acute lymphoblastic leukemia [2012 US] Disc. * 2022
Mepact^®^ (Mifamurtide)	IDM Pharma	Bone cancer [2009 EU]
Myocet^®^ (Doxorubicin)	Teva UK	Bone cancer [2000 EU]
Onivyde^®^ (Irinotecan hydrochloride)	Ipsen Biopharmaceuticals	Pancreatic cancer [2015 US] [2016 EU]
Vyxeos^®^ (daunorubicin)	Jazz Pharmaceuticals	AML, AML with myelodysplasia related changes [2017 US]
Onpattro^®^ (patisiran)	Alnylam	Hereditary transthyretin (TTR) mediated amyloidosis [2018 FDA and EMA]
Visudyne^®^ (Verteporfin)	Novartis	Age-related macular degeneration [2000 US/EU] Pathologic myopia [2000 US/EU] Ocular histoplasmosis [2000 US/EU]
Micellar Nanoparticle		
EstrasorbTM^®^ (Estradiol hemihydrate)	Novavax/Graceway	Menopausal therapy [2003 US] Disc. *
Taxol^®^ (Paclitaxel)	Bristol Myres Squibb	Ovarian cancer [1992 US] Breast cancer [1994 US] Disc. *
Taxotere^®^ (Docetaxel)	Sanofi-Aventis	Head and neck cancer [2006 US] [1995 EU]
Protein Nanoparticle		
Abraxane^®^ (ABI-007 Protein-bound paclitaxel)	Celgene	Breast cancer [2005 US] [2008 EU]
Ontak^®^ (Denileukin diftitox)	Eisai	Cutaneous T-cell lymphoma [1999 US]
Nanocrystals		
Avinza^®^ (Morphine sulfate)	Pfizer	Pain management [2002 US] Disc. *
Emend^®^ (Aprepitant)	Merck	Antiemetic [2003 US/EU]
Ivemend^®^ (fosaprepitant dimeglumine)	Merck	Antiemetic [2008 US/EU]
Focalin XR^®^ (Dexmethylphenidate hydrochloride)	Novartis	Attention deficit hyperactivity disorder [2005 US]
Invega Sustenna^®^/Xeplion^®^ (Paliperidone palmitate)	Janssen Pharms	Schizophrenia [2009 US] [2011 EU]
Zyprexa^®^ (Olanzapine)	Lilly Pharma	Schizophrenia [1996]
Megace ES^®^ (Megestrol acetate)	Endo Pharms	Anti-anorexic [2005 US]
Rapamune^®^ (Sirolimus)	Pfizer	Immunosuppresent [1999 US] [2001 EU]
Ritalin LA^®^ (Methylphenidate hydrochloride)	Novartis	Attention deficit hyperactivity disorder [2002 US]
Ryanodex^®^ (Dantrolene sodium)	Eagle Pharmaceuticals	Malignant hypothermia [2014 US]
Tricor^®^ (Fenofibrate)	AbbVie	High cholesterol and high triglyceride levels [2004 US]
Triglide^®^ (Fenofibrate)	SkyePharma AG	High cholesterol and triglycerides [2005 US]
Zanaflex^®^ (Tizanidine hydrochloride)	Acorda	Muscle relaxant [1996 US]
Inorganic and Metallic Nanoparticles		
Dexferrum^®^/ DexIron^®^ (iron dextran)	Luitpold Pharmaceuticals	Iron deficiency [1996 US] Disc. * 2014
FerahemeTM/Rienso^®^ Ferumoxytol (ferumoxytol)	AMAG pharmaceuticals	Iron deficiency anemia in chronic kidney disease [2009 US] [2012 EU]
Ferrlecit^®^ (iron carboxymaltose colloid)	Sanofi Avertis	Iron deficiency anemia in chronic kidney disease [1999 US]
INFeD^®^ (iron dextran)	Allergan Pharma	Iron deficiency anemia in chronic kidney disease [1974 US]
Injectafer^®^/Ferinject^®^ (iron carboxymaltose colloid)	Luitpold Pharmaceuticals	Iron deficiency anemia in chronic kidney disease [2013 US]
Hensify^®^ (hafnium oxide nanoparticles)	Nanobiotix	Locally advanced squamous cell carcinoma [2019 EU]
Venofer^®^ (iron sucrose)	Luitpold Pharmaceuticals	Iron deficiency anemia in chronic kidney disease [2000 US]
Nano-therm	MagForce	Recurrent glioblastoma, Prostate Cancer [2010 EU, 2018 US]
Dendrimer based Nanoparticles		
VivaGel^®^ BV (astodrimer sodium)	Starpharma	Anti-infective for prevention of recurrent bacterial vaginosis (BV) [2015 US]

* Disc.: discontinued.
